# Reduced White Matter Integrity With Cognitive Impairments in End Stage Renal Disease

**DOI:** 10.3389/fpsyt.2018.00143

**Published:** 2018-04-19

**Authors:** Yi Yin, Meng Li, Chao Li, Xiaofen Ma, Jianhao Yan, Tianyue Wang, Shishun Fu, Kelei Hua, Yunfan Wu, Wenfeng Zhan, Guihua Jiang

**Affiliations:** ^1^Guangdong Second Provincial General Hospital, Third School of Clinical Medicine, Southern Medical University, Guangzhou, China; ^2^Department of Medical Imaging, Guangdong Second Provincial General Hospital, Guangzhou, China

**Keywords:** end-stage renal disease, tract-based spatial statistics, white matter, cognitive function, serum creatinine, blood urea nitrogen

## Abstract

**Background:** End-stage renal disease (ESRD) is a serious public health problem, which can often lead to multiorgan dysfunction, such as cerebrovascular disease and cognitive damage. It is essential to understand cognitive impairment in patients with ESRD to develop better ESRD treatment and prevent further cognitive impairment. Cognitive impairment is believed to be related to structural abnormalities in the brain.

**Purpose:** To investigate white matter microstructural abnormalities in patients with ESRD using TBSS analysis of DTI and to explore the possible mechanisms underlying the impaired cognitive function.

**Materials and Methods:** A TBSS analysis of DTI data was to investigate the microstructural changes in their WM over the whole brain. We chose the white matter tracts or regions with significantly reduced FA as the regions of interest (ROIs), Pearson's correlations were performed between clinical indicators (Mini-Mental State Examination (MMSE), digit span task scores, serum creatinine, blood urea nitrogen and hemodialysis duration) and the mean FA value of the ROIs in the ESRD patients.

**Results:** Lower FA and higher MD, AD and RD values were observed in widespread and symmetrical WM in ESRD patients than healthy controls (HCs), Pearson correlation analysis revealed a significantly positive correlation between the Mini-Mental State Examination (MMSE) scores and FA values in the right corona radiata and left anterior thalamic radiation (ATR) and demonstrated a significantly negative correlation between FA values and the serum creatinine and blood urea nitrogen in the ATR (*P* < 0.01) in addition, digit span task scores positively correlate with the FA value in the left anterior rather than in the corona radiata. No cluster survived when we adopted the False Discovery Rate (FDR) correction to multiple comparisons.

**Conclusion:** Our study indicate widespread impairment of the white matter in ESRD patients. Damage to the thalamic radiation and corona radiata may affect cognitive function in ESRD patients, the reduced integrity of ATR may tend to affect the working memory while the damage to the corona radiata may involve the executive function impaired in ESRD patients. The accumulation of serum creatinine and blood urea nitrogen may contribute to the WM impairment.

## Introduction

End-stage renal disease (ESRD) is a serious public health problem [1, 2]. It occurs when glomerular filtration (GFR) falls below 15/mL/min/1.73 m^2^. In China, approximately 270,000 patients with ESRD must accept dialysis or renal transplantation therapy to sustain their lives, and both these treatments are extremely costly [3, 4]. ESRD can often lead to multiorgan dysfunction, such as cerebrovascular disease and cognitive damage [5]. Increasingly, attention has focused on cognitive damage because approximately 90% of ESRD patients exhibit impairment of cognitive function [6], which may profoundly affect the quality of their lives. Understanding cognitive impairment in patients with ESRD is important because it can be used to plan ESRD treatment and so prevent further cognitive impairment in the early stage.

In previous studies, participants with aggravated chronic kidney disease (CKD) (eGFR < 30) were more likely to have significant cognitive impairment, such as naming and attention [3, 7, 8, 14]. Abnormal brain functional connectivity was also observed in ESRD patients with resting-state functional MRI (r-fMRI) [9]. For example, an r-fMRI study found that ESRD patients exhibited significantly decreased functional connectivity with the posterior cingulate cortex (PCC) in the left middle temporal gyrus, the right anterior cingulate gyrus, and the bilateral medial superior frontal gyrus, which suggested a spatially specific disruption of functional connectivity of the default mode network (DMN) [10]. In addition to this fMRI study, there were also some structural abnormalities reported in ESRD patients, and some diffusion tensor imaging (DTI) studies detected changes in the WM in ESRD patients using several analytical methods, including region of interest (ROI) [9], voxel-based analysis (VBA) [11], and diffusion tensor tractographies (DTTs) [12], these studies found some microstructural abnormalities in ESRD patients, for example, the VBA study concluded that voxelwise DTI analysis is helpful in the detection of white matter alterations caused by hemodialysis. DTTs and r-fMRI detected structural and functional alterations in the DMN in the brain after renal transplantation in patients with ESRD [12]. This study showed the DMN may be damaged in the ESRD patients. However, this study was limited by its arbitrary choice of fibers for use with DTTs, which did not facilitate comprehensive assessment of microstructural changes in the WM. Although these studies showed WM microstructural changes in patients with ESRD, both the ROI and the DTTs methods are known to have low reproducibility due to the lack of a clear and consistent standard, and the results are dependent on the location and size of the ROI selected by the researcher. These smoothing and alignments are not accurate enough for VBA, which may affect the results. Tract-based spatial statistics (TBSS) is a recently developed DTI analytical method that require smoothness or a hypothesis [13–15], which renders it non-susceptible to the disadvantages of the conventional ROI or VBA-method [16]. To the best of our knowledge, a few studies have reported some white matter (WM) abnormalities using TBSS methods, for example, Kong et al. [14] found diffuse interstitial brain edema and moderate WM integrity disruption occurring in ESRD patients, which correlated with cognitive dysfunction, and serum urea levels might be a risk factor for these WM changes. Zhang et al. [13] found structural damages to radiation and associative fiber tracts may account for the cognitive deficits especially in executive function in ESRD patients. As we know, besides executive function, cognitive function involving working memory, and we found the working memory impaired in some ERSD patients in the neuropsychological tests. Recently, a study [18] indicated anterior thalamic radiation (ATR) abnormalities have a possible link with cognitive abnormalities (e.g., working memory) in schizophrenia. However, similar findings were not reported in patients with ESRD. The working memory may also impaired in ERSD patients, and the reduced integrity of ATR may related to the impaired working memory. The study has used TBSS methods to assess the structural abnormalities of the WM in ESRD patients.

The purpose of this study was to investigate microstructural changes in the white matter over the whole brain in ESRD patients using TBSS. Correlation analysis was performed between ESRD-related WM microstructural alterations and biochemical variables, digit span test scores and MMSE scores to detect the mechanism underlying cognitive impairment in ESRD patients.

## Materials and methods

### Subjects

This study was approved by the Research Ethics Review Board of the Institute of Mental Health at the Guangdong Second Provincial General Hospital, and written informed consent was obtained from each participant. The inclusion criteria of our study for the ESRD patients were as follows: (a) confirmed ESRD diagnosis according to the K/DOQI classification of CKD; (b) age between 20 to 60 years, female or male; (c) no history of kidney transplant or acute renal failure (ARF); (d) undergoing regular hemodialysis 3 times weekly at hospital and (e) right handedness. Exclusion criteria included a history of traumatic brain injury, psychiatric disease, or ischemic disease including acute ischemic cerebrovascular disease. Forty ESRD patients were recruited from July 2013 to June 2015. Among 40 patients, 4 with severe mental disorders or infarcts were excluded from the study, and one patient was excluded because of movement artifacts and poor image quality.

Finally, 35 ESRD patients (38.3 ± 10.9 years; 30 males) were enrolled in the present study. The duration of dialysis and renal disease were recorded from the patients' case histories. Here, 40 age-and sex-matched healthy controls (HCs) (41.6 ± 11.2 years; 33 males) were recruited in Guangzhou.

### Assessment of neurocognition

All participants underwent MMSE, digit span test and multiple biochemical tests before MR data acquisition. The digit span test is used to measure digital storage capabilities in working memory. Subjects can see or hear a series of numbers, and are responsible for correctly recalling the sequence of numbers. The sequence of numbers tested in each trial is longer. The criterion for digit span test score is the longest number of consecutive numbers that can be accurately remembered. The biochemical tests included Scr and BUN. The MMSE tests were performed by trained psychometricians.

### Image acquisition

MR Imaging was performed by using a 1.5T MR system (Achieva Nova-Dual; Philips) at the Department of Medical Imaging, Guangdong Second Provincial General Hospital with an eight-channel head coil. To exclude subjects with visible brain abnormalities, conventional imaging was performed using an axial T1-weighted image and T2-fluid attenuated inversion recovery (T2-FLAIR) image. DTI was performed with 32 diffusion gradient directions (*b* = 800 s/mm^2^ along 32 non-collinear directions) plus a reference image (i.e., *b* = 0) using a single shot spin echo planar sequence. The parameters were as follows: TR = 10,793 ms, TE = 62 ms, field of view = 230 × 230 mm^2^, matrix = 128 × 128, slice thickness = 2 mm, no slice gap, voxel size = 2 × 2 × 2 mm^3^.

### Data processing

All DTI images were processed using the PANDA toolbox (Version1.3.1, released 2016) based on the FSL [17]. Analysis of DTI parameters (MD, AD, and RD) was performed using TBSS implemented in FSL automatically. First, FA maps were calculated for all subjects from the DTI data after eddy-current-induced distortions and correct for head motion. Then, the FA data were spatially normalized into 1 × 1 × 1 mm^3^ Montreal Neurological Institute (MNI) 152 Space. Next, the mean FA (FA threshold > 0.2) image was created and thinned to create a mean FA skeleton. Finally, each subject's aligned FA data were projected onto this skeleton. MD, AD, and RD maps were also mapped onto the template using projection vectors from each individual's FA-to-skeleton transformation, and similarly conducted like FA images. After voxel-wise group comparisons, the skeletal regions showing significant differences were labeled using the JHU White-Matter Tractography Atlas in FSL.

The demographic, biochemical, and clinical characteristics were analyzed using the Statistical Package for the Social Sciences (SPSS) version 19.0 (SPSS Inc., Chicago, IL, US) and Student's *t*-tests were performed to compare the differences between the patients and the HCs with respect to age, education, and cognitive results. Gender differences were assessed using the Pearson Chi-Square test. The level of statistical significance was set at 0.05 (two tailed). Differences in diffusion indices between the ESRD group and HCs were assessed using voxel-wise two sample *t*-tests by FSL. Nonparametric permutation tests were conducted based on 5,000 random permutations. The clusters with a Threshold-Free Cluster Enhancement (TFCE) uncorrected *P*-value of less than 0.05 were reported, then we adopted the False Discovery Rate (FDR) correction to multiple comparisons.

### Outlining region-of-interest

We chose the white matter tracts or regions with significantly reduced FA as the regions of interest (ROIs), and then abstract the mean FA value of each ROIs, were delineated on FA images using Analyze AVW™ (Mayo Foundation, Rochester, Minnesota). b0 images are displayed with the FA image to help guide ROI delineation. ROIs were outlined by manually tracing the FA image and the position was confirmed on the b0 image. The same operator, who was unaware of the diagnostic team, tracked all ROIs. Mean FA value was computed to generate the total mean FA value for each ROIs.

### Correlation analysis

Pearson correlation analysis was performed between the mean FA value of each above significant clusters and the MMSE, digit span test, serum creatinine, serum urea levels, and duration of dialysis and disease respectively, we adopted the False Discovery Rate (FDR) correction to multiple comparisons.

## Results

### Demographic information and behavioral tests

The demographic, biochemical, and clinical characteristics for all the participants are shown in Table 1. There were no significant differences with respect to age, gender, or level of education between the ESRD patients and the healthy controls patients showed lower MMSE scores and digit span test score than healthy controls (*P* = 0.006) (*P* = 0.009).

**Table 1:** Demographic and clinical characteristics of ESRD patients and healthy controls.

**Variable**	**ESRD (*n* = 35)**	**HCs (*n* = 40)**	***P*-value**
Age (years)	38.3 ± 10.9	41.6 ± 11.2	0.117^a^
Gender (M/F)	30/5	33/7	>0.999^b^
Level of education (years)	11.3 ± 2.8	11.5 ± 2.5	0.153
Duration of dialysis (m)	16.3 ± 5.8	–	–
Duration of disease (m)	83.25 ± 33.65	–	–
Serum calcium (mmol/L)	2.5 ± 0.4	2.2 ± 0.3	0.513^b^
Serum creatinine (μmol/L)	826.3 ± 432.4	155 ± 228.6	< 0.001^b^
Blood urea nitrogen (mmol/L)	18.3 ± 8.5	5.3 ± 1.6	< 0.001^b^
MMSE score	25.69 ± 3.43	28.69 ± 5.13	0.006^b^
Digit span test score	7.3 ± 1.3	9.6 ± 1.6	0.009^b^

*Values are represented as mean ± standard deviation. ESRD, end-stage renal disease; HCs, healthy controls. MMSE, Mini-Mental State Examination*.

a *P-value was obtained by chi-square test*.

b *P-value was obtained by two-side two-sample t-test*.

### TBSS

**Figure 1:**
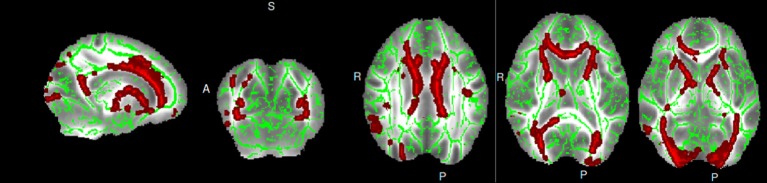
Inter-group differences for FA in ESRD patients and healthy controls.

**Figure 2:**
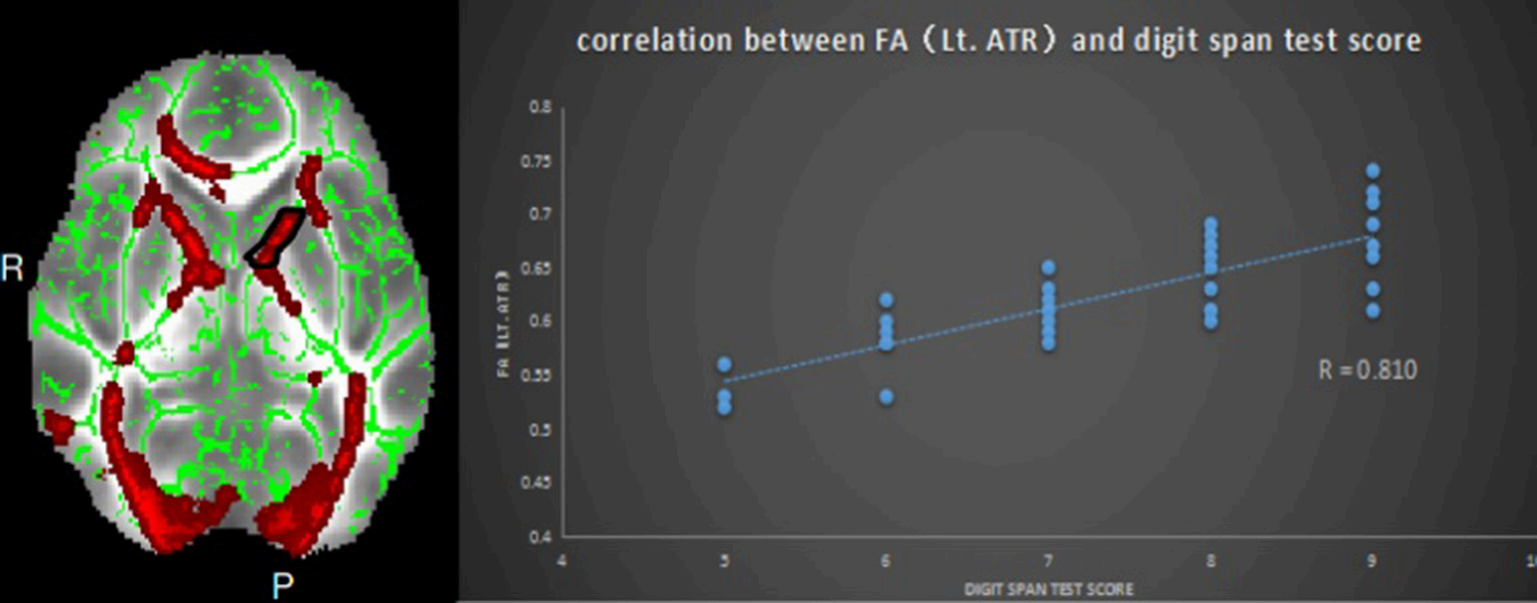
Relationship between mean FA and digit span test scores in ESRD; region-of-interest maps **(Left side)** are displayed with the corresponding graphs **(Right side)**. Significant positive correlation between FA values for the left anterior thalamic radiation with MMSE scores. Lt.ATR indicates left anterior thalamic radiation.

ESRD patients showed lower FA than HCs, mainly in bilateral corona radiata, bilateral ATR, bilateral inferior fronto-occipital fasciculus, the body and genu of the corpus callosum, bilateral superior longitudinal fasciculus (SLF), WM in the frontal lobe, and right inferior longitudinal fasciculus (Table 2; Figure 1). Specifically, widespread and symmetrical abnormal WM with increased MD, AD, and RD was observed in deep brain regions in ESRD patients, including corpus callosum, bilateral corticospinal tract, SLF, ATR, cingulate gyrus, superior temporal gyrus, and inferior prefrontal cortex (Figure 2). Unfortunately, we found no cluster survived when adopted the False Discovery Rate (FDR) correction to multiple comparisons.

**Table 2:** White matter tracts or regions with significantly reduced FA in patients and in healthy controls.

**Regions within the Brain**	**Side**	**MNI Coordinates (mm) (*x, y, z*)**	**Cluster Voxels**
Corona radiata	L	(−18, 40, 3)	2108
	R	(20, 42, 0)	2682
Anterior thalamic radiation	L	(−28, −69, 25)	1356
	R	(29, −65, 23)	1102
Inferiofronto-occipital fasciculus	L	(−35, −65, 2)	763
	R	(39, −36, 1)	473
Body of corpus callosum	N/A	(−11, 5, 33)	513
Genu of corpus callosum	N/A	(−13, 31, 13)	352
Superior longitudinal fasciculus	L	(−44, −10, 25)	231
	R	(43, −6, 26)	132
Inferior longitudinal fasciculus	R	(34, −65, −1)	84

*R indicates right, L, left, N/A, not applicable, MNI, Montreal Neurological Institute. All P < 0.05, uncorrected*.

### Correlation analysis

**Figure 3:**
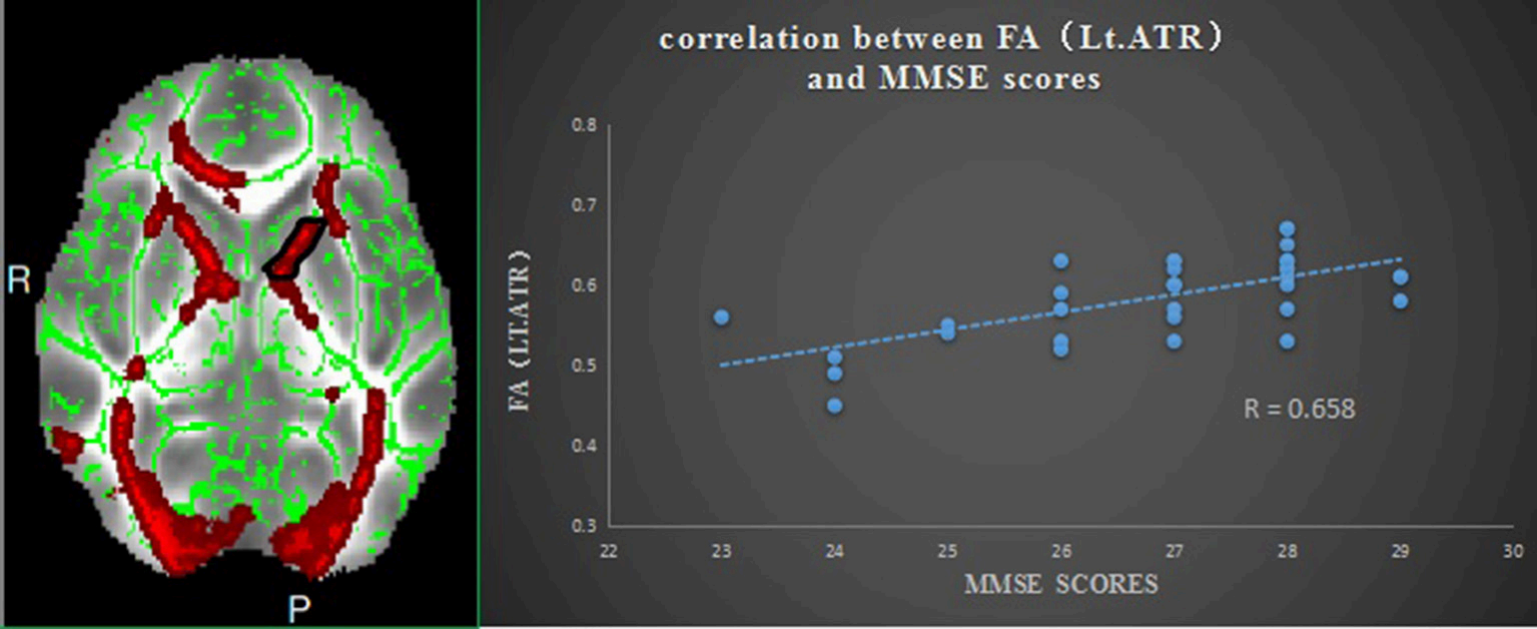
Relationship between mean FA and MMSE scores in ESRD; region-of-interest maps **(Left side)** are displayed with the corresponding graphs **(Right side)**. Significant positive correlation between FA values for the left anterior thalamic radiation with MMSE scores. Lt.ATR indicates left anterior thalamic radiation.

**Figure 4:**
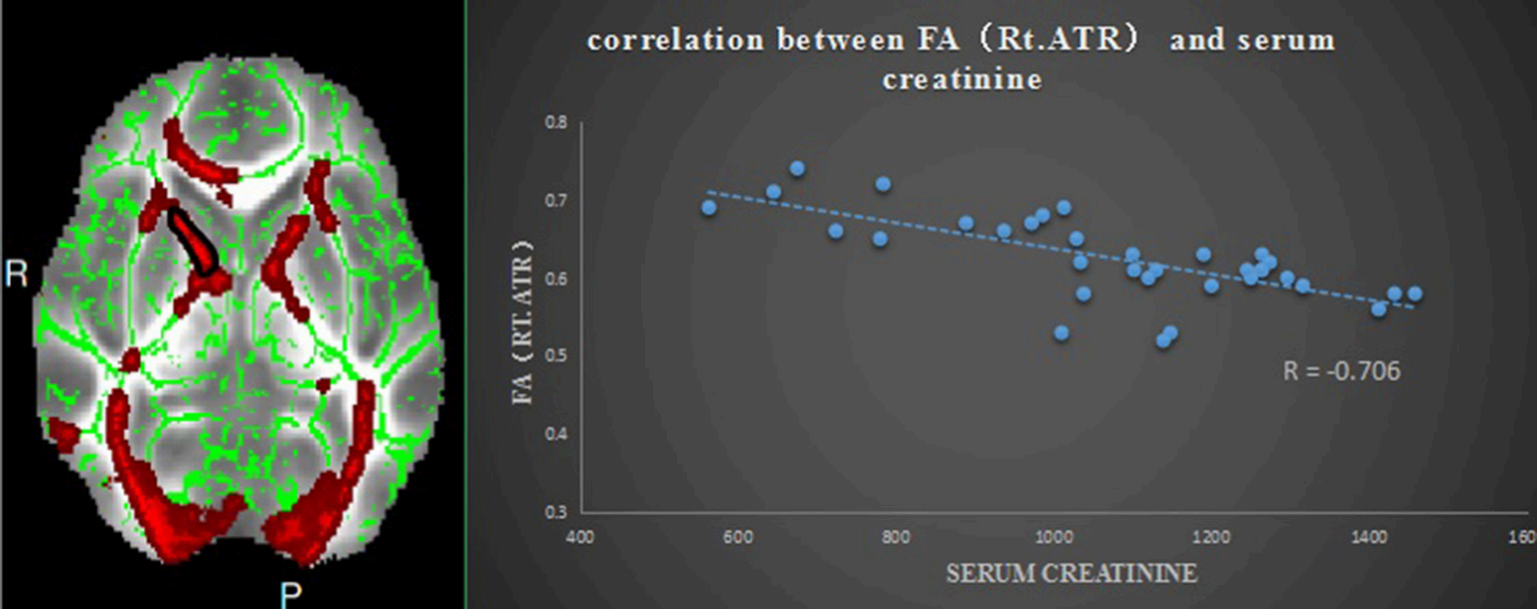
Relationship between mean FA and the serum creatinine in ESRD; region-of-interest maps **(Left side)** are displayed with the corresponding graphs **(Right side)**. Significant negative correlation between FA values for the right anterior thalamic radiation with the serum creatinine. Rt.ATR indicates right anterior thalamic radiation.

**Figure 5:**
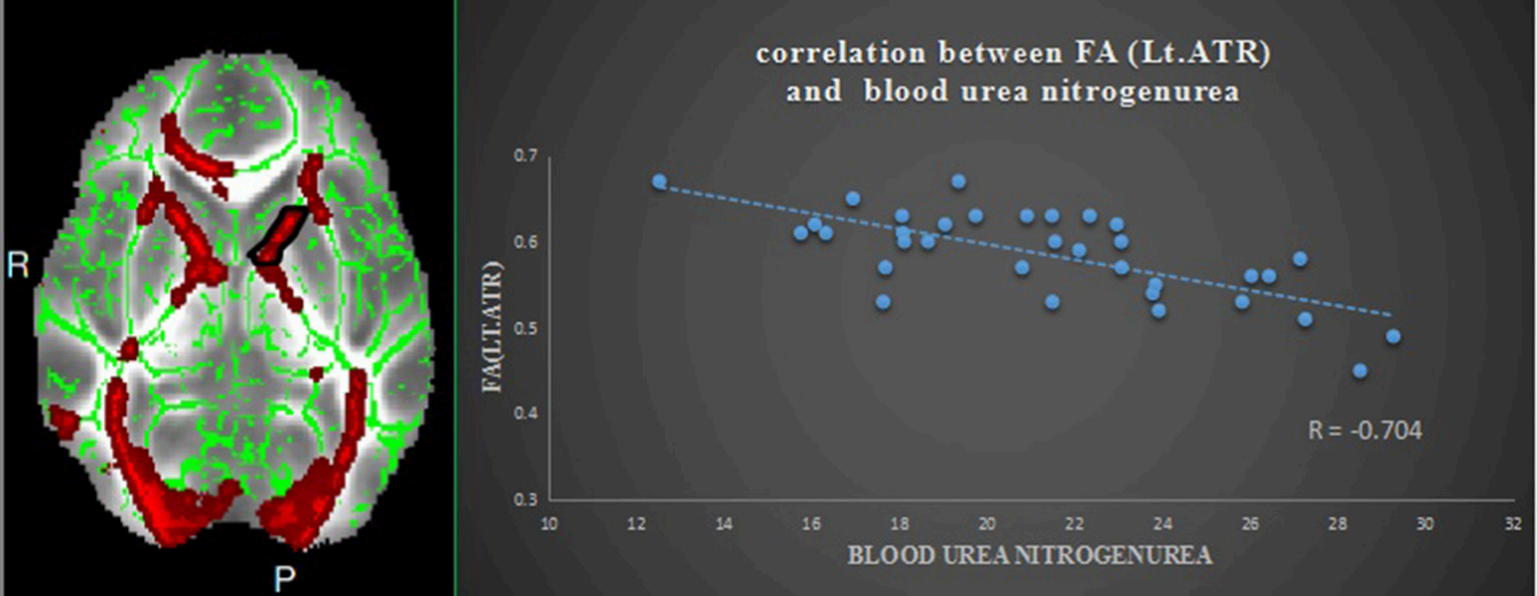
Relationship between mean FA and blood urea nitrogen in ESRD; region-of-interest maps **(Left side)** are displayed with the corresponding graphs **(Right side)**. Significant negative correlation between FA values for the left anterior thalamic radiation with blood urea nitrogen in ESRD. Lt.ATR indicates left anterior thalamic radiation.

Pearson correlation analysis revealed that the digit span test scores was positively correlate with the FA value in the left ATR (*r* = 0.810, *p* < 0.01, uncorrected) (Figure 2). And also revealed a significantly positive correlation between the MMSE score and FA values in the right corona radiata (*r* = 0.632, *P* < 0.01, uncorrected) and the left ATR (*r* = 0.658, *P* < 0.01, uncorrected) (Figure 3), and we found a significantly negative correlation between FA value and the serum creatinine in the right ATR (*r* = −0.706, *P* < 0.01, uncorrected) (Figure 4) and a significantly negative correlation between FA value and the blood urea nitrogen in the left ATR (*r* = −0.704, *P* < 0.01, uncorrected) (Figure 5) and the right corona radiata (*r* = −0.701, *P* < 0.01, uncorrected). We found no cluster survived when we adopted the False Discovery Rate (FDR) correction to multiple comparisons. There was no significant correlation between the MMSE scores and other non-FA indices and also show no significant correlation between FA value and sex, serum creatinine, serum urea levels, and duration of dialysis and disease (all *P* > 0.05).

## Discussion

We used TBSS to explore the integrity of WM in ESRD patients and to further examine the relationship between WM microstructure, MMSE scores, and biochemical indicators. ESRD patients showed decreased FA and increased MD, AD, and RD, mainly in the bilateral corona radiata and ATR, and the body and genu of the corpus callosum. Several structural abnormalities, such as the FA value in the left ATR, are negatively associated with the level of serum creatinine and blood urea nitrogen and positively associated with MMSE and digit span test scores.

The ESRD patients showed reduced FA in the ATR. Previous studies showed that the ATR is associated with memory encoding [18, 19]. In one study that performed on rats, working memory was found to be impaired after the thalamic radiation became damaged, so the thalamic radiation appears to be necessary to maintain the performance of working memory [20]. In another study [21], researchers found that the thalamic radiation is disproportionally larger and more complex in humans than in other mammals, adjusting for body size, which may contribute to humans' special cognitive abilities, such as working memory and language. This supports the hypothesis that reduced integrity of the ATR may affect the cognitive function of ESRD patients. The MMSE scores and digit span test scores were found to be positively correlated with FA values in the left ATR in ESRD patients. The performance of the digital span test is closely related to the working memory ability, and improving the language memory ability can help master the new language. Zhang et al. [13] indicated damages to the white matter may affect executive function in the ESRD patients, but didn't report that the working memory was impaired. Our results indicated the working memory was also impaired in ESRD patients. One possible explanation for the impaired working memory in ESRD patients is that the anterior thalamic nuclei process afferent information from the hippocampus, which is involved in cognitive functions such as working memory, and they project mainly toward the anterior cingulate cortex. Reduced integrity of the ATR may disrupt this process, and the ATR is thought to consist of default mode network (DMN) [22–24]. DMN is thought to be involved in the core processes of human cognition, including the integration of cognitive and emotional processing, mind wandering, and monitoring of the surrounding environment [24–27]. Harciarek et al. found that the DMN recovered shortly after renal transplantation in ESRD patients [28]. In this way, the reduced integrity of the ATR in our study suggested that the working memory is impaired in ESRD patients.

We also found decreased FA values in the corona radiata, which was consistent with previous studies. For example, one study performed using the VBA-method showed that the FA value of the bilateral corona radiata was decreased in ESRD patients [11]. Another study suggested that structural damages to the corona radiata may disrupt neural transmission and affect the central executive network [29]. The decreased FA value in the corona radiata suggests it may affect the executive function in ESRD patients. The corona radiata is an important group of nerves because of its role in the relationship between regions in the brain. It contains afferent nerves that send messages of sensory input from the body to the brain, and the efferent nerves send motor control function messages from the brain to the body [30]. This means that the corona radiata carries messages to and from the body, and the reduced microstructure integrity observed in the corona radiata in this study indicate the impaired function of sensory input function and motor control in ESRD patients.

In addition, ESRD patients showed decreased FA and increased MD, AD, and RD, mainly consistent with the white matter regions that FA reduced. MD, AD, and RD reflect the overall or direction-specific capability of tissue water diffusion. These indices usually increase when WM undergoes demyelination, axon loss, or some other process. This suggested that decreased FA may be caused by both increased AD and increased RD, indicating that both demyelination and axonal injury may lead to the WM impairment in ESRD patients, which is partially consistent with previous DTI studies [8, 31]. Using DTI, Zhang et al. showed significant increases in RD values but no significant changes in AD values [13]. Another study, which used VBA, showed both increased RD and AD values in some WM regions [11], but the results showed no consistent relationship to abnormal brain structure in patients with ESRD. Hsieh found that the FA value in patients with ESRD was significantly reduced compared to the control group in the whole brain region by using manual region-of-interest analysis [32]. The FA value generally reduce in the elderly and long-term hemodialysis patients. This may be related to degeneration of axons and demyelination of white matter. The decrease of FA and the increase of MD may attributed to macroscopic tissue damage and the destruction of the microstructural integrity caused by interstitial edema. Moreover, they found the positive correlation between MD and RD values and duration of dialysis suggests that dialysis may be a factor in white matter demyelination. Long-term dialysis leads to interstitial edema and demyelination leading to pons of axons. They believe that dialysis has a broad impact on the white matter structure of patients with ESRD. Interstitial edema of patients with ESRD in dialysis may explain cognitive impairment in patients with ESRD [14]. However, our study didn't found any relationship between dialysis duration and FA, MD, AD, and RD value. One possible reason could be that the sample difference, which may affect the result. The other possible reason could be the heterogeneity of subjects due to the lack of more rigorous subject recruitment. A further study with a larger sample of subjects is needed to address this issue in the future. In addition, there is reason to believe that, in addition to dialysis may lead to brain cognitive impairment, patients with ESRD itself can also damage the brain structure and thus affect cognitive function due to renal failure caused the accumulation of toxic substances.

Finally, this study shows the positive correlation of serum creatinine and blood urea nitrogen with the reduced FA in the bilateral ATR. One previous study [29] suggested that serum creatinine and blood urea nitrogen may cause reduced WM integrity. One possible explanation for this is that renal function damage may lead to accumulation of metabolic agents, and these neural toxicities could lead to the demyelination and axonal injury [33]. Some studies [6, 34] have shown that serum creatinine and blood urea nitrogen are associated with cognitive dysfunction in patients with ESRD, and neuropsychologic performance has been shown to improve after renal transplantation. In a recent resting-state fMRI study, Bai et al. [31] reported similar findings in patients with ESRD and observed that metabolic agents were associated with altered functional connectivity in patients with ESRD. Our results indicate that serum creatinine and blood urea nitrogen may contribute to the cognitive impairment. It is essential to take steps to control the level of serum creatinine and blood urea nitrogen to prevent further impairment of cognitive function in ESRD patients.

The present work has several limitations that must be considered. First, the MRI scanning parameters were suboptimal (e.g., 1.5-T scanner) for the current dataset. Second, we only observed patients at the end stage of choric kidney disease because chronic kidney disease can be classified into 5 stages, and it progresses to ESRD when the glomerular filtration rate (GFR) falls below 15 ml/1.73 m^2^, more studies should be performed in the future to explore cognitive impairment in CKD before the end stage because this cognitive impairment is mild and may reverse if detected and treated promptly. Finally, we found no cluster survived if we adopted the False Discovery Rate (FDR) correction to multiple comparisons. One possible reason could be that the sample size was not large enough in our study (35 patients), which may affect the statistical power, the other possible reason could be the heterogeneity of subjects due to the lack of more rigorous subject recruitment. A further study with a larger sample of subjects is needed to address this issue in the future.

In conclusion, the results of our study indicate the reduced integrity of the corona radiata and ATR may be related to cognitive function in ESRD patients. the impaired cognitive function involve not only in executive function but also involving the working memory. In addition, from a pathological perspective, this study indicates that the accumulation of metabolic agents such as serum creatinine and blood urea nitrogen may contribute to cognitive impairment in ESRD patients. These findings may facilitate understanding of the relationship between WM microstructural abnormalities and physiological alterations in ESRD. Although the patho-physiology is complicated, and there may be many factors that affect this WM impairment, this study provides an effective approach for exploring WM abnormalities in ESRD patients.

## Author contributions

GJ: Designed experiments; XM, JY, and TW: Carried out experiments; CL: Analyzed experimental results; KH, SF, and YW: Analyzed sequencing data and developed analysis tools. WZ: Assisted with collecting data; YY and ML: Wrote the manuscript.

### Conflict of interest statement

The authors declare that the research was conducted in the absence of any commercial or financial relationships that could be construed as a potential conflict of interest.
